# The effect of calcium supplementation on calcium and bone metabolism during load carriage in women: protocol for a randomised controlled crossover trial

**DOI:** 10.1186/s12891-023-06600-w

**Published:** 2023-06-16

**Authors:** Charlotte V. Coombs, Sophie L. Wardle, Rukshana Shroff, Anton Eisenhauer, Jonathan C. Y. Tang, William D. Fraser, Julie P. Greeves, Thomas J. O’Leary

**Affiliations:** 1 Army Health and Performance Research, Army Headquarters, Andover, SP11 8HT UK; 2 Renal Unit, UCL Great Ormond Street Hospital for Children NHS Foundation Trust and Institute of Child Health, London, UK; 3 GEOMAR Helmholtz Centre for Ocean Research, Kiel, Germany; 4 Norwich Medical School, University of East Anglia, Norwich, UK

**Keywords:** Bone metabolism, Calcium, Supplement, Exercise, Load carriage

## Abstract

**Background:**

Military field exercises are characterised by high volumes of exercise and prolonged periods of load carriage. Exercise can decrease circulating serum calcium and increase parathyroid hormone and bone resorption. These disturbances to calcium and bone metabolism can be attenuated with calcium supplementation immediately before exercise. This randomised crossover trial will investigate the effect of calcium supplementation on calcium and bone metabolism, and bone mineral balance, during load carriage exercise in women.

**Methods:**

Thirty women (eumenorrheic or using the combined oral contraceptive pill, intrauterine system, or intrauterine device) will complete two experimental testing sessions either with, or without, a calcium supplement (1000 mg). Each experimental testing session will involve one 120 min session of load carriage exercise carrying 20 kg. Venous blood samples will be taken and analysed for biochemical markers of bone resorption and formation, calcium metabolism, and endocrine function. Urine will be collected pre- and post-load carriage to measure calcium isotopes for the calculation of bone calcium balance.

**Discussion:**

The results from this study will help identify whether supplementing women with calcium during load carriage is protective of bone and calcium homeostasis.

**Trial registration:**

NCT04823156 (clinicaltrials.gov).

## Background

Military training is physically arduous [1] and involves prolonged periods of heavy load carriage [2]. The demands of military training [1] and load carriage [2] are greater in women than men, and women have a greater incidence of stress fracture [3]. These sex differences are predominantly due to the typically lower average aerobic fitness, strength, and muscle mass of women than men when completing the same tasks [1–3]. To maintain calcium homeostasis, blood calcium concentrations are tightly regulated [4]. Strenuous exercise disturbs calcium homeostasis; a reduction in serum calcium increases parathyroid hormone (PTH) and markers of bone resorption to maintain circulating concentrations of calcium [5–7]. Exercise intensity and duration influence the magnitude of the exercise-induced disturbance to calcium homeostasis [8]. In young women, load carriage exercise decreased serum ionized calcium, increased PTH, and increased fractional calcium absorption [9]. Calcium supplementation before exercise can mitigate the exercise-induced disturbances to calcium homeostasis, as shown in a series of walking and cycling studies [8]. Military training and field exercise place significant mechanical and metabolic demands on the skeleton [10–14], and the decrease in bone mineral density (BMD) in the axial skeleton and increase in BMD in the appendicular skeleton [15, 16] may be indicative of disturbed calcium homeostasis and/or insufficient calcium intake. A daily calcium supplement (2000 mg·d^− 1^) during military training prevented an increase in PTH [17], augmented tibial adaptations [5, 7], and reduced the incidence of stress fractures [18]. However, the effect of calcium supplementation before an acute bout of military load carriage on calcium and bone metabolism in women is unknown.

Biochemical markers of bone resorption and formation are inherently variable and only infer the metabolic state of bone turnover at the precise instance at which they are measured. Biochemical markers of bone resorption and formation often increase or decrease together and cannot be used to determine overall net bone mineral balance (*i.e.* whether bone resorption or bone formation is the prevailing process) [19]. Skulan and DePaolo (1999) [20] were the first to outline a conceptual model describing the fractionation of calcium isotopes between soft tissue and bone. Differences in isotopic composition between soft tissue and bone were reported, with bone being enriched with isotopically lighter calcium isotopes compared to soft tissue [20, 21]. Decreased BMD is associated with a net loss of calcium from the bone. Using the model formulated by Skulan and DePaolo (1999) [20], measuring changes in the natural calcium isotopic composition of calcium in the urine provides a non-invasive method to determine calcium homeostasis and calcium flux to, and from, bone [22–25]. Six, naturally occurring, stable calcium isotopes exist in the diet: ^40^Ca, ^42^Ca, ^43^Ca, ^44^Ca, ^46^Ca, and ^48^Ca. Bone formation favours the uptake of isotopically lighter calcium isotopes causing a shift in urine composition towards an isotopically heavier state [26]. When bone resorption is dominant, and calcium is lost from bone, the isotopic calcium composition of the urine becomes lighter [27]. Low calcium intake results in a negative bone calcium balance (*i.e.* calcium is derived from the skeleton to maintain serum calcium concentrations) [28], but the effects of military load carriage and calcium supplementation on bone calcium balance, determined from circulating and urinary calcium isotopes, is unknown.

This study will investigate the effect of calcium supplementation on calcium and bone metabolism in response to load carriage exercise (standard military fitness test; 2 h, 20 kg, 6.4 km·h^− 1^) in military women, a population at increased risk of stress fracture during military training compared with men [3]. It is hypothesised that calcium supplementation will attenuate the decline in serum ionized calcium and increase in PTH and bone resorption, and prevent a negative calcium balance during load carriage. A secondary aim will investigate the effect of load carriage on quadricep and hamstring strength. It is hypothesised that quadricep and hamstring strength will be lower following load carriage.

## Methods

**Fig. 1:**
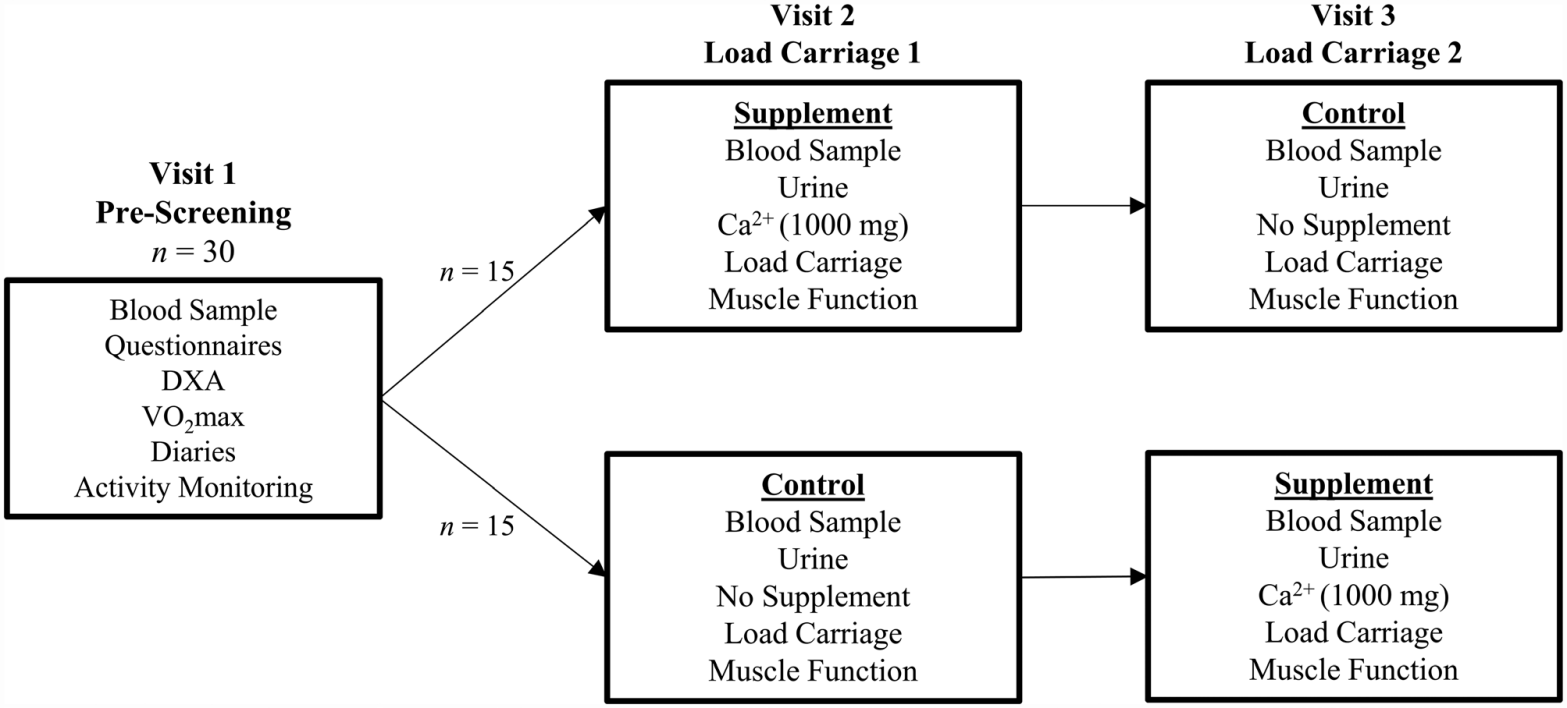
Overview of the study design. Ca^2+^, calcium; Questionnaires: physical activity readiness questionnaire, menstrual history questionnaire, and Eating Attitudes Test – 26 item; Diaries: exercise and menstrual; DXA, dualenergy X-ray absorptiometry; $\dot V$O_2_max, maximal rate of oxygen uptake.

This study is a non-blinded randomised crossover controlled trial. Following the completion of a screening visit to confirm study eligibility (Pre-Screening; visit 1), each participant will complete a single load carriage exercise session on two separate occasions (visits 2 and 3) completed in a randomised order; one with a calcium supplement (Supplement) (1000 mg) and one without (Control) (Fig. 1). Block randomisation using a block size of 2 will be performed and there will be no allocation concealment. All laboratory testing will be completed at the Army Human Performance Laboratory based at the Royal Military Academy, Sandhurst, UK.

### Participants

Thirty women will complete this randomised controlled crossover trial. Participants will: be aged 18 to 36 years old; have a maximal rate of oxygen uptake ($\dot V$O_2max_) of ≥ 35 mL·kg^−1^·min^−1^; will be eumenorrhoeic or using the combined oral contraceptive pill, an intrauterine system, or intrauterine device; be weight stable, defined as no change in self-reported body mass ≥ 5% over the previous 3 months; have a BMI between 18 and 30 kg·m^−2^, and; not be pregnant or lactating. Women will be excluded if they: have disordered eating, defined as ≥ 20 on the Eating Attitudes Test – 26 item (EAT-26); are vitamin D deficient, defined as a total 25-hydroxyvitamin D (25(OH)D) < 30 nmol·L^−1^; have a total areal BMD T-score of < − 1; are oligomenorrhoeic or amenorrhoeic (< 9 menstrual cycles in previous 12 months); self-report as currently smoke or vape or have stopped smoking within the previous three months; are taking any medication known to affect bone or calcium metabolism (*e.g.* treatment for thyroid disorders); have a history of heart, liver, or kidney disease, diabetes, or thyroid disorder; have had a stress fracture or other bone injury in the previous 12 months, or; are anaemic (haemoglobin < 12 g·dL^−1^).

### Recruitment

Regular and Reserve military servicewomen, and female civilians, will be invited to volunteer. Individuals interested in particpating will contact the investigators, upon which they will recieve a Participant Information Sheet and be invited to a phone conversation. The study aims, requirements, right to withdraw, and risks will be outlined. This study has received favourable ethical opinion from the Ministry of Defence Research Ethics Committee (1021/MoDREC/19). During this study participants will therefore be covered by the Ministry of Defence for compensation under the provisions of the no-fault compensation scheme.

### Pre-screening

Consent to participate will be obtained from all volunteers by the investigators. Participants will have the option to consent for any remaining samples to be retained for follow-up analyses related to calcium metabolism, bone health, and endocrine function.

#### Height and body mass

Height will be measured using a stadiometer (Seca, UK) and body mass will be measured using calibrated weighing scales (Seca, UK) with participants wearing a T-shirt and shorts.

#### Questionnaires and activity monitoring

Participants will be asked to complete an adjusted physical activity readiness questionnaire [29], a menstrual history questionnaire [30], and the EAT-26 questionnaire [31, 32]. Participants will be asked to declare any current medication and dietary supplement use, including hormonal contraceptives, and previous history of stress fracture. Participants will be issued a menstrual diary to record down menses dates until the end of their participation in the study to aid scheduling of load carriage sessions. Habitual exercise energy expenditure (kcal) will be measured over seven days following the pre-screen visit from raw accelerometery data during periods of purposeful exercise recorded in an exercise diary [33]. Accelerometery data will be obtained from a wrist-worn device (GENEActiv, Activinsights, UK) worn for 24 h per day and sampled at 100 Hz [33]. Purposeful exercise will be defined as training/competition sessions, including leisure or transport activities (*e.g.* running, cycle to work, hiking, swimming, military physical training).

#### Blood sample

A single venous blood sample will be drawn into vacutainers from a vein in the antecubital fossa for the measurement of haemoglobin, serum total 25(OH)D for vitamin D status and intact PTH (iPTH) [29].

#### Dual-energy X-ray absorptiometry (DXA)

Participants will initially complete a pregnancy test to confirm they are not pregnant. A whole-body scan (Lunar iDXA™, GE Healthcare) will be performed with participants lying supine on the scanning table. Participants will be asked to wear shorts and a T-shirt and remove any jewellery. Whole-body and regional areal BMD, T-score, fat mass, fat-free mass, and lean mass will be determined from the scan.

#### Maximal rate of oxygen uptake

An incremental maximal exercise treadmill test (ELG, Woodway, USA) will be performed to determine $\dot V$O_2max_ [34]. Participants will run on the treadmill at a starting speed of 5 km·h^−1^ with the speed increased by 1 km·h^−1^ per minute until volitional exhaustion. Expired gases (Metalyzer 3B, Cortex, Germany) and heart rate (H10, Polar, Finland) will be measured continuously. Maximal rate of oxygen uptake will be taken as the highest 30 s average $\dot V$O_2_. Where $\dot V$O_2max_ criteria is not attained (34) $\dot V$O_2peak_ will be reported.

### Load carriage 1 and 2

Participants who meet the inclusion criteria from pre-screening will complete two independent load carriage exercise sessions in the laboratory separated by a minimum of two weeks. Participants not taking any hormonal contraceptives will track their menstrual cycle with the provided menstrual diary and start each experimental testing session in the late lutal phase/early follicular phase (within the few days just before or after menstruation). Participants using the combined oral contraceptive pill will complete each experimental testing session during the seven day break between pill packets; participants taking back-to-back combined oral contraceptive pill packets, using an intrauterine system, or intrauterine device will be tested at any point.

**Fig. 2:**
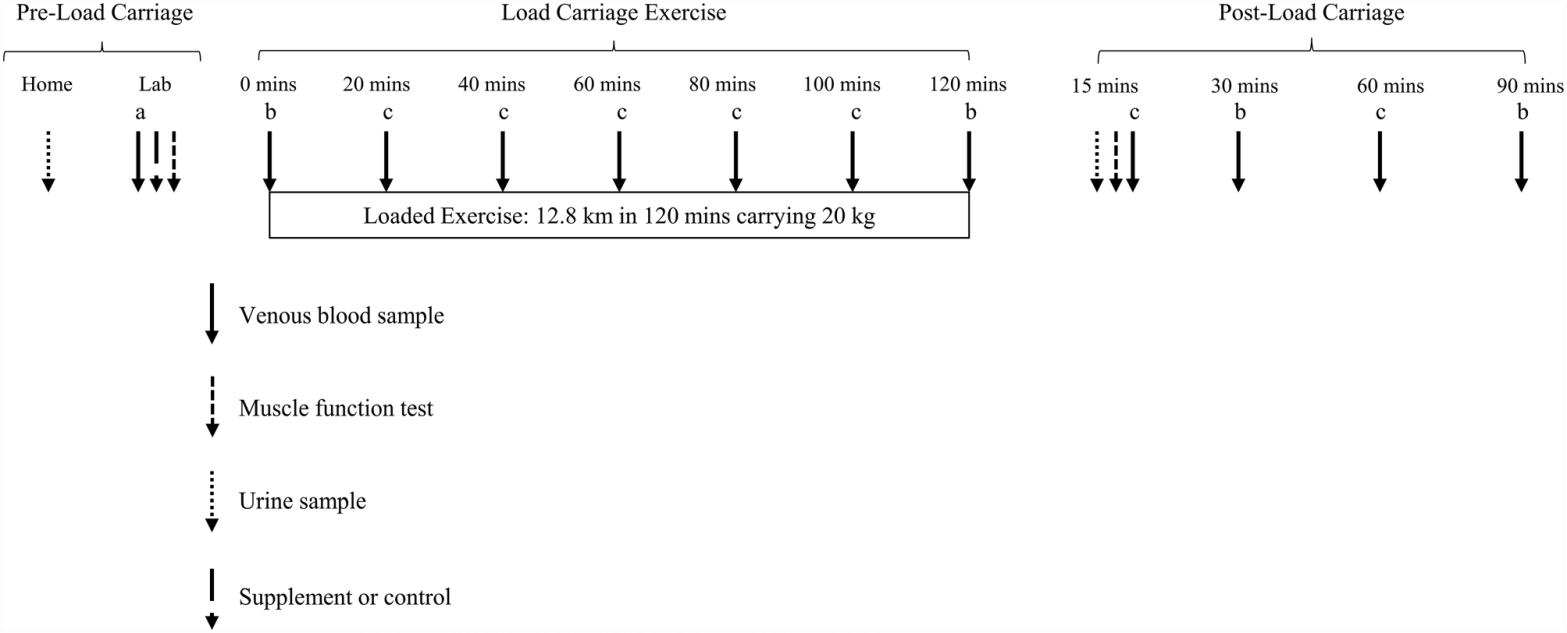
Overview of load carriage exercise testing protocol. ^a^Procollagen type 1 N-terminal propeptide, bone-specific alkaline phosphatase, beta carboxy-terminal cross-linking telopeptide of type 1 collagen, albumin-adjusted calcium, ionized calcium, ^44^Ca:^42^Ca, phosphate, intact parathyroid hormone, 25-hydroxyvitamin D, 1,25-dihydroxyvitamin D, 24,25-dihydroxyvitamin D, luteinising hormone, follicle stimulating hormone, oestradiol, testosterone, and sex hormone binding globulin will be measured before load carriage. ^b^Procollagen type 1 N-terminal propeptide, beta carboxy-terminal cross-linking telopeptide of type 1 collagen, ionized calcium, albumin-adjusted calcium, phosphate, intact parathyroid hormone, sclerostin and osteocalcin will be measured at 0 and 120 min during load carriage, then 30 and 90 min after load carriage. ^44^Ca:^42^Ca will be measured at 120 min during load carriage. ^c^Procollagen type 1 N-terminal propeptide, beta carboxy-terminal cross-linking telopeptide of type 1 collagen, ionized calcium, albumin-adjusted calcium, phosphate, and intact parathyroid hormone will be measured at 20, 40, 60, 80 and 100 min during load carriage, and 15 and 60 min after load carriage.

In the 24 h preceding each load carriage session participants will be rested (no exhaustive exercise) and will not apply skin moisturizer. Participants will arrive at the laboratory after an overnight fast, have an indwelling cannula inserted in a vein in the antecubital fossa, and a resting blood sample taken (Fig. 2). A calcium supplement (2 × 500 mg; Calcichew) will be given to each participant completing the Supplement condition ~ 60 min before starting the load carriage exercise [8]. Sweat collectors (Macroduct Sweat Collector, ELITech Group, France) [35] will be attached to the forearm for the measurement of sweat calcium excretion during exericse. A 120 min session of load carriage exercise will be completed. The load carriage exercise is a fitness test completed by the infantry. Participants will walk 12.8 km on the treadmill at 6.4 km·h^− 1^ for 120 min whilst wearing a 20 kg military backpack. Expired gases, heart rate, and rating of perceived exertion will be measured throughout the exercise. The first urine void upon waking (Fig. 2), and a urine void within ~ 1 h of the cessation of load carriage, will be obtained for the measurement of calcium isotopes for the calculation of bone calcium balance. Nude body mass will be measured before and immediately after exercise for the determination of sweat loss and calculation of sweat calcium excretion; post-exercise body mass will be adjusted for water consumed *ad libitum* during exercise. Calcium concentration of water consumed during exercise will be recorded. Blood samples will be taken at 0 min, and then every 20 min during, and 15, 30, 60, and 90 min following, load carriage. Blood samples fractioned into serum and plasma will be analysed for markers of bone formation, bone resorption, and calcium metabolism. Whole blood will be analysed immediately for blood lactate concentration (Lactate Pro 2, Arkray, Japan), ionized calcium, haemoglobin and haematocrit (i-STAT Alinity, Abbott, USA; CHEM8^+^, Abbott, USA) (Fig. 2). Isokinetic (60°·s^− 1^ and 180°·s^− 1^) and isometric (90° knee flexion) maximal voluntary contraction of the knee extensors and flexors (System 4, Biodex Medical Systems, USA) will be measured before and immediately after load carriage exercise. Researchers will monitor the laboratory testing procedures to ensure adhereance to study protocols; there is no criteria for the study to be discontinued.

### Biological sample collection and storage

Venous blood samples will be drawn into vacutainers for the collection of serum (SST II Advance serum separator, and silica, Becton Dickinson, USA) and plasma (K2EDTA, Becton Dickinson, USA). Serum and plasma vacutainers will stand at room temperature for 30 min before centrifugation at 3000 *g* 4 °C for 10 min. Serum and plasma fractions will be stored at −80 °C until analysis. Venous blood samples for the collection of whole blood will be drawn into a lithium heparin vacutainer (Becton Dickinson, USA), analysed immediately and then disposed of. Urine voids will be collected into a 1 L container; a 10 mL volume will be aspirarted into a Monovette (Sarstedt, UK) containing boric acid and stored at −80 °C until analysis. Sweat samples will be transferred to 1.5 mL microvettes and stored at −80 °C until analysis.

### Calcium isotope measurement in blood, urine, and sweat

Blood (serum), urine, and sweat samples will be analysed for total calcium and the stable calcium isotopes ^42^Ca and ^44^Ca as described elsewhere [24]. Urine (1 mL) and serum (0.25 mL) will be digested in 14 mol/L HNO_3_ and 2 mL of H_2_O_2_ (30%), then heated to 220 °C for 45 min and dried overnight at 120 °C. Dried samples will be dissolved in 2 mol/L HNO_3_ to reach a calcium concentration of 25 mg/L then undergo automatic chromatographic purification (prepFAST MC, ESI, USA); a solution almost solely containing calcium will be collected with a yield of more than 85%. Calcium solutions will be dried and treated at 120 °C for 3 h with 1 mL 14 mol/L, l HNO_3_ and 0.5 mL of H_2_O_2_ (30%), then dissolved in 0.2 mol/L HNO_3_ to yield a concentration of 2.5 mg/L. A maximum of 21 samples, one blank, and one of each reference material (NIST SRM 915a, IAPSO Standard Seawater, NIST SRM 1486), and one in-house standard for urine (AK-1) or serum (SERA-1) will be collected. Calcium isotope measurements will be performed on a Multicollector-Inductively Coupled Plasma-Mass Spectrometer (MC-ICP-MS) (Neptune plus, Thermo Fisher Scientific, Bremen, Germany). The mass spectrometer is equipped with nine Faraday cups of which eight are moveable. The mass spectrometer is set up to measure masses 42, 43, 43.5, and 44 simultaneously. To prevent interfering calcium and argon hybrids (*e.g. *^40^Ar^1^H_2_ on ^42^Ca) an APEX IR (ESI, Omaha, Nebraska, USA) sample introduction system is used. All measurements will be performed in medium resolution (MR, m/Δm ~ 4,000) on the interference-free plateau of the low mass side of the peaks, achieved by choosing an appropriate center cup mass of 43.687 ± 0.001 amu, verified on a daily basis (cf. [36]). Instrumental fractionation (mass bias) will be corrected by applying the standard-sample-bracketing approach. The measurement of a sample will be bracketed by measurements of a ~ 5 µg·mL calcium solution prepared from a 10,000 µg·g calcium ICP reference solution. Every sample will be measured at least four times during a session and the mean value used for further calculations. There is negligible isotope fractionation during purification (< 0.01‰) with this method. Although almost all strontium is removed from the samples during chemical preparation, samples will be monitored for doubly charged strontium (^84^Sr, ^86^Sr, and ^88^Sr) that can interfere with the measurement of ^42^Ca, ^43^Ca, and ^44^Ca (cf. [37]). Several criteria will be applied to reject data of a single measurement, a single sample, or a whole sequence [37]: a single sample will be rejected when |δ^44/42^Ca – 2·δ^43/42^Ca|>0.2‰; a single sample (4 or 5 single measurements) will be rejected when the average intensity is outside a 70 to 130% intensity window when compared to the average intensity of the reference solution from the same batch; a whole sequence will be rejected when more than one of the measured international reference materials deviates more than 0.2‰ from the literature value or the data will not fall along the mass-dependent fractionation line. The long-term 2-sigmal standard deviation reproducibility of the Ca isotope value is 0.06‰.

### Biochemical markers of bone formation and bone resorption

Pre-screen and load carriage (Fig. 2) blood sample analysis will be performed at the Bioanalytical Facility, University of East Anglia (Norwich, UK) by the COBAS automated platform (Roche Diagnostics, Mannheim, Germany). Procollagen type 1 N-terminal propeptide, (P1NP), iPTH, beta carboxy-terminal cross-linking telopeptide of type 1 collagen (βCTX) and osteocalcin will be analysed in EDTA plasma, whereas albumin-adjusted calcium, phosphate, luteinising hormone, follicle stimulating hormone, oestradiol, and sex hormone binding globulin in serum. Sample analysis will be carried out according to manufacturers’ instructions and under Good Clinical and Laboratory Practice conditions. The inter-assay coefficient of variations (CV) are ≤ 3% within their respective analytical working range. LC-MS/MS will be used to measure serum testosterone, 25(OH)D3, 25(OH)D2, 24,25-dihydroxyvitamin D3 (24,25(OH)_2_D3), 24,25-dihydroxyvitamin D2 (24,25(OH)_2_D2), 1,25-dihydroxyvitamin D3 (1,25(OH)_2_D3), and 1,25-dihydroxyvitamin D2 (1,25(OH)_2_D2), with their respective vitamin D metabolite ratio [38]. The measurement ranges of the assays are: 0–25 nmol/L for 24,25(OH)_2_D2 and 24,25(OH)_2_D3, and 15–600 pmol/L for 1,25(OH)_2_D2 and 1,25(OH)_2_D3. The mean CV for intra-assay imprecision across the measuring range of the assays are: ≤ 7.7% for 24,25(OH)_2_D2, 9.0% for 24,25(OH)_2_D3, and ≤ 7.4% for 1,25(OH)_2_D. The cumulative inter-assay CVs are: ≤ 10.6% for 24,25(OH)_2_D2, ≤ 8.9% for 24,25(OH)_2_D3, and ≤ 9.3% for 1,25(OH)_2_D. Plate-based enzyme-linked immunosorbent assay (ELISA) will be used to analyse for bone-specific alkaline phosphatase (bone ALP) (MicroVue, Quidel, San Diego, CA, USA) and sclerostin (Biomedica, Vienna, Austria) in serum. Inter-assay CV for bone ALP is ≤ 5.8%, and  < 8.5% for sclerostin.

### Data analyses

Data management will be conducted by the investigators. All data will be pseudo anonymised, and double data entry and value range checks will be performed. The investigators will collect and assess the outcome measurements. Exercise energy expenditure measured with raw accelerometery data [33] during periods of purposeful exercise, and recorded in the exercise diary, will be processed with the GENEAread R package from CRAN [39] using a cusomized, openly available script [33, 40]. The ratio of ^44^Ca to ^42^Ca (^44^Ca:^42^Ca) in urine, blood and sweat will be measured following published standard procedures [24, 25]. The calcium isotopic composition is reported as δ^44/42^Ca in parts per thousand (‰):

$$\begin{array}{l}{\delta ^{44/42}}Ca{\rm{ }}\left( {{\% _0}} \right){\rm{ }} = {\rm{ }}\\\left[ {{{\left( {^{44}Ca{/^{42}}Ca} \right)}_{Sample}}/{{\left( {^{44}Ca{/^{42}}Ca} \right)}_{Reference}}} \right]{\rm{ - }}1\end{array}$$

Change between pre- and post-load carriage urine samples will be calculated for each load carriage session.

### Statistical analyses

No previous studies have been published investigating calcium balance through natural calcium isotope ratios in response to exercise or calcium supplementation, therefore the required sample size for this study is calculated based upon data from high and low calcium diets to reflect the Control (no calcium) and Supplement (1000 mg calcium) conditions within this study. The required sample size was calculated in R (version 4.1.1) using the *‘pwr’* package based on testing the null hypothesis that there will be no difference in calcium balance between Control and Supplement conditions. Based on differences in calcium balance between women on a high (~ 1500 mg·d^− 1^) and low (~ 300 mg·d^− 1^) calcium diet (108.6 ± 127.9 vs. − 95.2 ± 24.6 mg·d^− 1^, d = 2.21) [28], and men on a low (376 mg·d^− 1^) and moderate (857 mg·d^− 1^) calcium diet (10.0 ± 6.0 vs. 0.1 ± 1.8 parts per ten thousand, d = 2.24) [22], it is anticipated that 5 people per group will be required to detect a difference in calcium balance between the Control group and Supplement group with an α of 0.05 and a 1 – ß of 0.90. The data from these trials were using chronic diets rather than acute supplementation and so we anticipate that the effect we will see will be much smaller. Therefore, a second sample size calculation was performed from PTH concentration (a secondary outcome) in response to exercise. Based on a 18.86 to 26.83 pg·mL^− 1^ [41–43] higher serum PTH during exercise without calcium supplementation compared with calcium supplementation (following a calcium meal [1352 ± 53 vs. 46 ± 7 mg [42]] or calcium drink [1000 mg·L^− 1^ [41]; 486 mg·L^− 1^ [43]), it is anticipated that 26 people will be required to detect a difference in serum PTH concentration (d = 0.667) between the Control group and Supplement group with an α of 0.05 and a 1 – ß of 0.90. A total sample of 30 will allow us to detect an effect of d ≥ 0.75 with an α of 0.01 and a 1 – ß of 0.90.

A per-protocol analysis will be performed. Significance will be accepted as p < 0.05.

#### Primary outcomes

The absolute change in urine calcium balance (^44^Ca:^42^Ca) from pre- to post-load carriage will be compared between the Control condition and Supplement condition using a one-way analysis of covariance (ANCOVA) with pre-load carriage calcium balance (either as a ratio, or both the numerator and denominator) as the covariate. The minimal important difference in absolute change in urine calcium balance between the Control condition and Supplement condition is not known.

#### Secondary outcomes

Circulating measures of calcium and bone metabolism during load carriage will be compared between Control and Supplement conditions, and changes in knee flexor and extensor strength will be compared between pre- and post-load carriage, using linear mixed models with the restricted maximum likelihood estimation to allow incorporation of incomplete data. Time and group (Control or Supplement) will be assigned as fixed effects and participant will be assigned as random effects to account for within participant correlation for repeated measures. Within exercise data will be compared between trials using paired sample *t*-tests.

## Discussion

Military women are at increased risk of stress fracture during military training compared with men [3], and strategies to protect the skeletal health of this population are required. Women in the UK Armed Forces are exposed to heavy prolonged periods of load carriage. Load carriage exercise has been found to increase PTH, decrease serum ionized calcium, and increase fractional calcium absorption [9]. The effect is likely intensity dependent with higher intensity exercise causing the rise in PTH [44], which appears to make women more susceptible to PTH increases during basic military training [14]. Acute calcium supplementation can attenuate acute disturbances in calcium homeostasis during exercise [45], but whether a similar effect is present in military load carriage is unknown. The results from this study will help identify whether supplementing women with calcium during load carriage is protective of calcium and bone metabolism. Understanding the influence of load carriage on calcium and bone homeostasis, and whether the provision of a calcium supplement attenuates exercise-induced disruption to calcium homeostasis, will improve our understanding of the effect of military activities on skeletal metabolism and support evidence-based nutritional policies. Energy supplementation during military field exercises and operations are often ineffective in eliminating energy deficits [46] and other solutions are required to protect bone health in energy deficits. These data will have important utility in other physically arduous occupations and in female athletes. Data from this study will be presented to the Ministry of Defence and published where possible.

## Data Availability

The data that support the findings of this study will be available from The Ministry of Defence but restrictions apply to the availability of these data, which were used under license for the current study, and so are not publicly available. However data will be available from the authors upon reasonable request and with permission of The Ministry of Defence.

## Abbreviations

25(OH)D: 25-hydroxyvitamin D
ANCOVA: analysis of covariance
βCTX: beta carboxy-terminal cross-linking telopeptide of type 1 collagen
Bone ALP: bone-specific alkaline phosphatase
BMD: bone mineral density
BMI: body mass index
Ca^2+^: calcium
CV: coefficient of variation
DXA: dual-energy X-ray absorptiometry
EAT-26: Eating Attitudes Test – 26 item
ELISA: enzyme-linked immunosorbent assayu
iPTH: intact parathyroid hormone
LC-MS/MS: liquid chromatography tandem mass spectrometry
MC-ICP-MS: Multicollector-Inductively Coupled Plasma-Mass Spectrometer
P1NP: procollagen type 1 N-terminal propeptide
PTH: parathyroid hormone
$\dot V$O_2max_: maximal rate of oxygen uptake
$\dot V$O_2peak_: peak oxygen uptake
$\dot V$O_2_: volume of oxygen per minute
